# Mucor and Malignancy: A Double-Hit Case of Intestinal Ischemia

**DOI:** 10.14309/crj.0000000000000907

**Published:** 2022-12-19

**Authors:** Benjamin T. Berger, Matthew J. Townsend, Mitchell K. Arbogast, Jenny Van Kirk

**Affiliations:** 1 Department of Medicine, Duke University Medical Center, Durham, NC; 2 Department of Pathology, Duke University Medical Center, Durham, NC

## Abstract

Mucormycosis is an invasive fungal infection due to molds in the order *Mucorales*. These opportunistic pathogens found in soil or decaying organic matter mostly affect immunocompromised hosts. Rhino-orbital-cerebral, pulmonary, gastrointestinal, cutaneous, and disseminated patterns are possible. We describe a case of angioinvasive colonic mucormycosis in a patient with recent diabetic ketoacidosis and undiagnosed colon adenocarcinoma. The diagnosis was made on histopathology after the patient developed intestinal ischemia and underwent hemicolectomy. This case highlights the potentially diverse manifestations of *Mucorales* infections, typical and atypical risk factors, and the index of suspicion necessary for early diagnosis and outcome optimization.

## INTRODUCTION

Mucormycosis describes invasive infections by molds in the order *Mucorales*, typically *Rhizopus* and *Mucor* species.^1^ These molds are ubiquitous in the environment, especially in soil and decaying organic matter.^2^ They produce spores to which humans are exposed through inhalation, ingestion, or inoculation. Risk factors for *Mucorales* infections include solid organ or bone marrow transplantation, hematologic malignancy, neutropenia, corticosteroid use, trauma, and diabetes mellitus, particularly with diabetic ketoacidosis (DKA).^3,4^ Recently, coronavirus disease 2019 (COVID-19) has been identified as a risk factor of mucormycosis, possibly because of immunomodulatory effects of the virus and steroids used in its management.^5,6^ Although mucormycosis most commonly presents with rhino-orbital-cerebral involvement, primary gastrointestinal mucor represents approximately 7%–8% of mucormycosis cases.^1,7^ Treatment for any pattern includes surgical source control and polyene antifungal therapy. Mortality ranges from 25% (isolated sinus disease) to >90% (disseminated disease).^1^ We describe a case of angioinvasive gastrointestinal mucormycosis presenting with colonic ischemia in a patient with recent DKA and undiagnosed colon adenocarcinoma.

## CASE REPORT

A 77-year-old woman presented with 2 weeks of epigastric pain, nausea, and poor oral intake. Medical history was notable for long-standing type 2 diabetes mellitus poorly controlled on metformin (A1c 10.7%). She had a metabolic acidosis (pH 7.02, bicarbonate 6 mEq/L), hyperglycemia (862 mg/dL), and elevated beta-hydroxybutyrate consistent with DKA. Aspartate and alanine transaminases, alkaline phosphatase, and total bilirubin were elevated at 148, 370, 658 U/L, and 7.7 mg/dL, respectively. The patient underwent abdominal ultrasound and computed tomography (CT), which demonstrated duodenal thickening concerning for duodenitis but did not reveal an etiology of her cholestasis. Her DKA resolved with intravenous insulin and fluids. She was transferred to our medical center for advanced gastroenterological evaluation.

**Figure 1.**
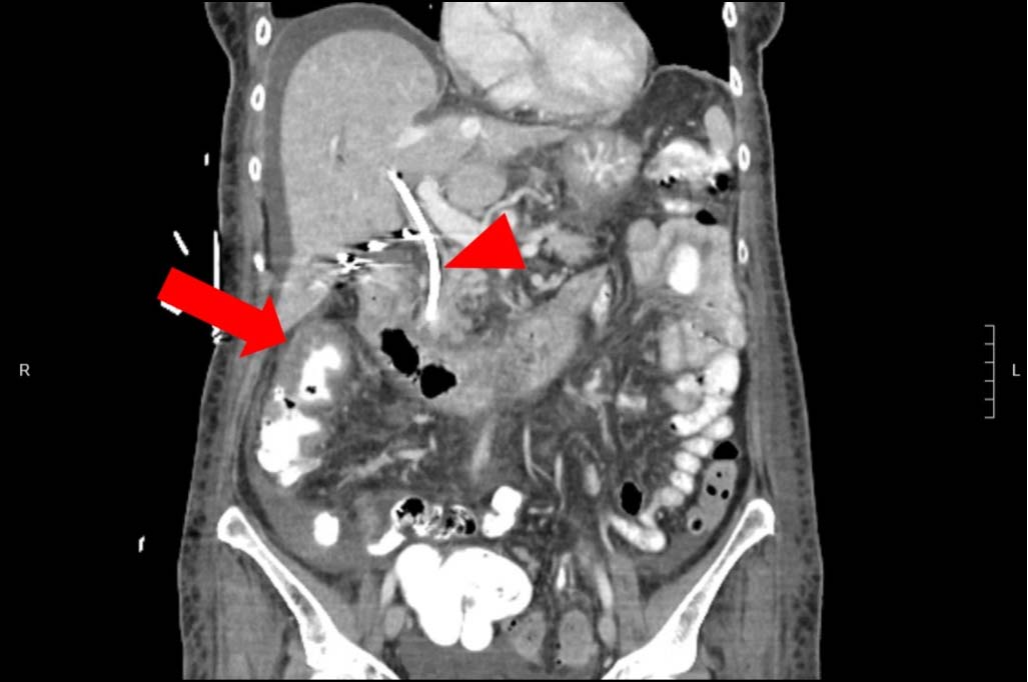
Abdominal and pelvic computed tomography with intravenous and oral contrast showing segmental wall thickening of the ascending colon (arrow) consistent with colitis. The biliary stent is also visualized (arrowhead).

On transfer, physical examination demonstrated upper abdominal tenderness without signs of peritonitis. Laboratory studies showed leukocytosis and persistent liver enzyme elevations in a cholestatic pattern. The patient was treated with piperacillin-tazobactam and vancomycin for possible cholangitis. Magnetic resonance cholangiopancreatography showed luminal irregularities and dilation of the left and right intrahepatic bile ducts with focal narrowing of the common hepatic duct and wall thickening of the common bile duct. Subsequent endoscopic retrograde cholangiopancreatography showed malignant-appearing strictures of the common hepatic and common bile ducts. Brushings were obtained, and the common bile duct was stented. Pathology of bile duct brushings revealed an abnormal ductal epithelium suspicious for adenocarcinoma. On postprocedure day 2, the patient had multiple maroon bowel movements. Mesenteric CT angiography showed new colonic wall thickening but did not identify active bleeding or thrombosis. The patient developed worsening abdominal pain and leukocytosis, and interval abdominal CT displayed worsening ascending colonic inflammation (Figure 1).

**Figure 2.**
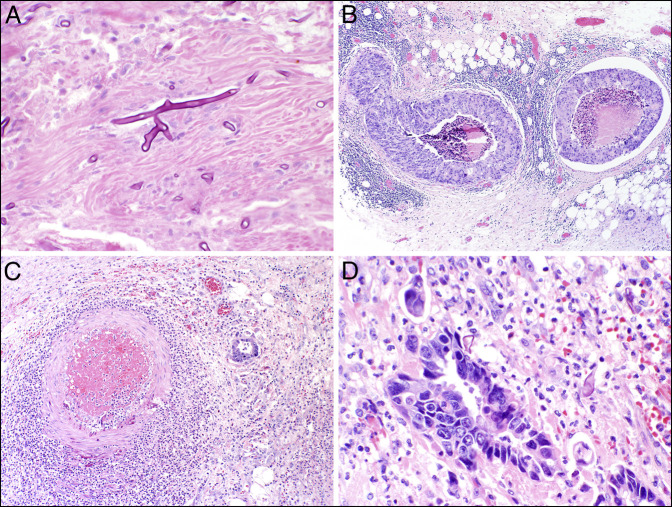
Representative histologic photomicrographs. (A) Broad nonseptate fungal hyphae with a 90° angle branching involving the muscular wall of the colon, morphologically consistent with mucormycosis (H&E ×40). (B) Intravascular colonic adenocarcinoma involving large mesenteric veins (H&E ×4). (C) Angioinvasive fungus involving a mesenteric artery with associated thrombus formation and an adjacent focus of adenocarcinoma (H&E ×10). (D) High-power view of invasive adenocarcinoma colliding with fungal hyphae (H&E ×40). H&E, hematoxylin and eosin.

The patient underwent diagnostic laparoscopy, which revealed a necrotic ascending and transverse colon with perforation. Right hemicolectomy and end ileostomy were performed. Echocardiogram demonstrated normal cardiac function. The colitis was presumed to be ischemic in etiology, although no preceding hypotension had been observed, and there was no evidence of thromboembolism. On postprocedure day 12, histopathology of the resected colon revealed angioinvasive mucormycosis (Figure 2). In addition, an occult adenocarcinoma was identified with extensive lymphovascular invasion and lymph node metastases. Fungal elements and neoplastic cells were seen in close association (Figure 2). Her presenting cholestasis was consistent with malignant biliary obstruction from this primary colon adenocarcinoma.

Liposomal amphotericin B was initiated for mucormycosis. Evaluation of other sites of involvement was pursued with head magnetic resonance imaging, which showed no evidence of rhino-orbital-cerebral disease, and CT chest, which showed nonspecific tree-in-bud nodularity of the upper lobes. The patient developed progressive renal dysfunction on amphotericin and was transitioned to isavuconazole. Her course was complicated by worsening malnutrition and anasarca. She decided to pursue hospice care and unfortunately died 1 month after discharge.

## DISCUSSION

Gastrointestinal mucormycosis carries high morbidity and mortality. While incidence varies globally, country-level data suggest a rising incidence of mucormycosis—for example, from 0.7 to 1.2 per million in France from 1997 to 2006—which may reflect increased detection and/or risk factors including diabetes mellitus (450–720 cases per million globally in the same period).^8-10^ The stomach is the most common site of gastrointestinal involvement, followed by the colon and ileum.^1,7^ Angioinvasion is a hallmark pathogenic feature that causes thrombosis and ischemia. Accordingly, gastrointestinal mucor can present with abdominal pain, nausea, vomiting, and gastrointestinal bleeding; bowel perforation is common in advanced disease. Bowel wall thickening or pneumatosis may be observed on imaging.^11,12^ Because symptoms and imaging are nonspecific and serologic testing is limited, diagnosis remains challenging and relies on histopathology.^13^ In one series, only 25% of gastrointestinal mucormycosis cases were identified antemortem.^1^ Early identification of risk factors may facilitate timely diagnosis.

Our patient's best established risk factor was poorly controlled diabetes with recent DKA. She also presented with malnutrition likely secondary to her later-diagnosed malignancy, which has been associated especially with the development of gastrointestinal mucormycosis.^1^ Although nosocomial outbreaks from colonized materials have been described, endoscopic equipment contamination is unlikely in our patient because her ischemic symptoms began within 48 hours after endoscopic retrograde cholangiopancreatography. Colon adenocarcinoma likely acted synergistically in the development of invasive mucormycosis. The extensive intravascular tumor observed on pathology contributed to ischemia, creating a local hypoxic and acidic tissue microenvironment in which *Mucorales* thrives.^14^ Iron content is increased in colon tumor tissue, and *Mucorales* are well known for their iron avidity.^15,16^

Treatment of mucormycosis relies on source control and antifungal therapy.^17^ Surgical intervention and early initiation of amphotericin B are associated with improved survival.^18,19^ *Mucorales* is most reliably sensitive to amphotericin B, but isavuconazole and posaconazole are used as step-down or salvage therapy. Antifungal therapy is complicated by susceptibility variations between and within *Mucorales* species, drug toxicities, poor correlation between in vitro and in vivo activity, and tissue ischemia, which impedes delivery to the infection site(s).^3,19^ Gastrointestinal mucormycosis should trigger evaluation for rhino-orbito-cerebral, pulmonary, cutaneous, or disseminated mucor; in our patient, the duodenitis and pulmonary tree-in-bud opacities observed on CT imaging may have represented multiorgan disease. This case should encourage clinicians to remain vigilant for mucormycosis, an increasingly prevalent infection with diverse clinical manifestations and high mortality that can be improved with prompt recognition and treatment.

## DISCLOSURES

Author contributions: BT Berger and MJ Townsend wrote the manuscript and reviewed the literature. MK Arbogast provided histopathology images. MK Arbogast and J. Van Kirk revised the manuscript for intellectual content. J. Van Kirk is the article guarantor.

Financial disclosure: None to report.

Informed consent was obtained for this case report.
